# Outcome at two years of age in a Swiss national cohort of extremely preterm infants born between 2000 and 2008

**DOI:** 10.1186/1471-2431-12-198

**Published:** 2012-12-28

**Authors:** Luregn J Schlapbach, Mark Adams, Elena Proietti, Maude Aebischer, Sebastian Grunt, Cristina Borradori-Tolsa, Myriam Bickle-Graz, Hans Ulrich Bucher, Beatrice Latal, Giancarlo Natalucci

**Affiliations:** 1 Neonatal and Paediatric Intensive Care Unit, Department of Paediatrics, University of Bern, Bern, Switzerland; 2 Paediatric Critical Care Research Group, Mater Children’s Hospital, Brisbane, Australia; 3 Department of Neonatology, University Hospital Zurich, Zurich, Switzerland; 4 Division of Pediatric Pneumology, University Children Hospital, Bern, Switzerland; 5 Division of Neonatology, Department of Paediatrics, University of Bern, Bern, Switzerland; 6 Division of Neuropaediatrics, Development and Rehabilitation, University Children’s Hospital, Berne, Switzerland; 7 Department of Paediatrics, Geneva University Hospital, Geneva, Switzerland; 8 Division of Neonatology, Department of Paediatrics, Centre Hospitalier Universitaire Vaudois, Lausanne, Switzerland; 9 Child Development Centre, University Children’s Hospital Zurich, Zurich, Switzerland

**Keywords:** Development, Disability, Mortality, Outcome, Preterm

## Abstract

**Background:**

While survival rates of extremely preterm infants have improved over the last decades, the incidence of neurodevelopmental disability (ND) in survivors remains high. Representative current data on the severity of disability and of risk factors associated with poor outcome in this growing population are necessary for clinical guidance and parent counselling.

**Methods:**

Prospective longitudinal multicentre cohort study of preterm infants born in Switzerland between 24^0/7^ and 27^6/7^ weeks gestational age during 2000–2008. Mortality, adverse outcome (death or severe ND) at two years, and predictors for poor outcome were analysed using multilevel multivariate logistic regression. Neurodevelopment was assessed using Bayley Scales of Infant Development II. Cerebral palsy was graded after the Gross Motor Function Classification System.

**Results:**

Of 1266 live born infants, 422 (33%) died. Follow-up information was available for 684 (81%) survivors: 440 (64%) showed favourable outcome, 166 (24%) moderate ND, and 78 (11%) severe ND. At birth, lower gestational age, intrauterine growth restriction and absence of antenatal corticosteroids were associated with mortality and adverse outcome (p < 0.001). At 36^0/7^ weeks postmenstrual age, bronchopulmonary dysplasia, major brain injury and retinopathy of prematurity were the main predictors for adverse outcome (p < 0.05). Survival without moderate or severe ND increased from 27% to 39% during the observation period (p = 0.02).

**Conclusions:**

In this recent Swiss national cohort study of extremely preterm infants, neonatal mortality was determined by gestational age, birth weight, and antenatal corticosteroids while neurodevelopmental outcome was determined by the major neonatal morbidities. We observed an increase of survival without moderate or severe disability.

## Background

Advances in perinatal care have resulted in improved survival rates of extremely preterm infants over the last three decades [1]. In contrast, the incidence of major neonatal diseases causing significant morbidity in this population remains unchanged [2]. Long-term outcome studies indicate higher vulnerability in a wide spectrum of developmental domains, ranging from somatic growth, learning abilities, behaviour, and motor performance to sensorial domains [3-8]. As a consequence, the high proportion of infants surviving with long-term neurosensory disabilities is cause of major concern. Gestational age, birth weight, sex, multiple birth, antenatal corticosteroid administration, neonatal infection, necrotizing enterocolitis (NEC), bronchopulmonary dysplasia (BPD), and major brain lesions such as periventricular leukomalacia (PVL) and intraventricular haemorrhage (IVH) have been shown to influence both short- and long-term outcome [9-11]. The analysis of outcomes in this population represents a crucial aspect of quality control and may identify risk factors that could potentially be targeted by specific intervention measures. In addition, decisions on the provision of intensive versus palliative care and counselling of parents of extremely preterm infants are based on the expected incidence of mortality and poor long-term outcome. Therefore, it is of paramount importance that representative and recent outcome data are available [10,12-14]. The present study aimed to describe the outcomes of a recent Swiss cohort of extremely premature infants at two years of age and to establish the main risk factors to predict mortality, and adverse and favourable outcome.

## Methods

### Study population

This was a prospective longitudinal multicentre cohort study including extremely premature infants live born between 24^0/7^ and 27^6/7^ weeks in Switzerland from January 1^st^ 2000 to December 31^st^ 2008. Routine follow-up of infants below 28^0/7^ weeks gestational age has been recommended and performed by the Swiss Neonatal Follow-up Group since 2000, and follow-up data are prospectively collected in the network database. After the recommendation was issued to base follow-up examinations on the Bayley Scales of Infant Development II (BSID-II) [15], not all centres were able to implement this immediately. Therefore, only infants from centres routinely assessing BSID-II were included for the analysis. Infants with major congenital malformations (defined as genetic anomaly, syndrome, or malformation of a major organ system) were excluded. Clinical and follow-up data for this study were prospectively recorded in the national database of the Swiss Neonatal Network & Follow-up Group. Data collection and evaluation for this study were approved by the institutional ethical review boards and by the Swiss Federal Commission for Privacy Protection in Medical Research. Participating centres were obliged to inform parents about the scientific use of anonymized data.

### Definition of neonatal variables

Gestational age was the best estimate available from the obstetric measurements based on last menstrual period or prenatal ultrasound findings, as recorded in the maternal chart. Birth weight z-scores were calculated based on the growth curves by Voigt et al. [16]. Major brain injury was defined as IVH grade 3 or higher according to Papile classifications [17] and/or presence of cystic PVL [18] on cerebral ultrasound. Bronchopulmonary dysplasia (BPD) was defined as requirement for additional oxygen at 36^0/7^ weeks postmenstrual age (PMA) [19]. Retinopathy of prematurity (ROP) was defined using the International Committee criteria [20]. NEC was defined as pneumatosis intestinalis or pneumatosis vena portae (Bell’s stage two or higher) [21]. The presence or absence of infection was classified into uninfected, suspected (clinical and laboratory signs of sepsis but absence of positive blood or cerebrospinal fluid culture, in an infant who received treatment with antibiotics for ≥ 5 days or until death), and proven sepsis (positive blood or cerebrospinal fluid culture) [22]. Socioeconomic status (SES) was estimated by a validated 12-point socioeconomic score based on maternal education and paternal occupation and was classified into higher class (score 2–5), middle class (6–8) and lower class (9–12) [23].

### Neurodevelopmental assessment

Neurodevelopmental examination was routinely performed by experienced developmental paediatricians or neuropaediatricians at each Swiss Neonatal Follow-up centre at 18–24 months corrected age (i.e. the age the infant would be if he had been born on his due date). The assessment consisted of a clinical examination, a structured neurological assessment and a developmental assessment using the BSID-II [15]. Vision and hearing were assessed either by direct examination or caregiver report. If a structured BSID-II testing could not be performed due to lack of cooperation, the exam was repeated 3 to 6 months later. Infants who were so severely impaired that a structured testing with the BSID-II could not be performed were assigned a mental development index (MDI) and psychomotor development index (PDI) of 49. Cerebral palsy (CP) was defined as a permanent disorder of movement and posture, causing activity limitations that are attributed to non-progressive disturbances that occurred in the developing fetal or infant brain [24] and was graded according to the Gross Motor Function Classification System of Palisano and associates for children aged ≤ 2 years [25].

### Outcome definitions

Outcome at two years of age was defined according to the guidelines of the working group of the British Association of Perinatal Medicine and the National Neonatal Audit Project on the Classification of Health Status [26]:

i) *Death* before two years of age.

ii) *Severe neurodevelopmental disability*; defined as CP with GMFCS level 3–5; or a BSID-II score of < −3SD below the mean (i.e. MDI or PDI <55), or absence of useful hearing even with aids (i.e. >90dB hearing level), or blindness or only perception of light or light reflecting objects.

iii) *Moderate neurodevelopmental disability*; defined as CP with GMFCS level 2, or a BSID-II score between −3 and −2 SD below the mean (i.e. MDI or PDI 55–69), or hearing loss corrected with aids (40-90dB hearing level), or moderately reduced vision but better than severe visual disability, or unilateral blindness with good vision in the contralateral eye.

iv) *Favourable outcome*; defined as absence of any of the above.

*Adverse outcome* was defined as the combination of death or severe ND. *Unfavourable outcome* was defined as the combination of death, or severe or moderate ND.

### Statistical analysis

Chi-square test and Mann–Whitney U test were used to compare subgroups in nonadjusted comparisons.

Two different time points were chosen for the outcome prediction models:

i) at time of birth: this dataset included all live born children, and the covariates known at birth

ii) at 36^0/7^ weeks PMA: this dataset included all infants surviving up to 36^0/7^ weeks PMA, and the covariates known by 36^0/7^ weeks PMA.

We first selected the clinical and demographic variables known to determine mortality and adverse outcome based on evidence from the literature [8,27]. We then checked these associations in our population using univariate logistic regression analysis. This was performed both in all infants being born and in all infants surviving to 36^0/7^ weeks PMA. Pre-planned sensitivity analyses were performed excluding children who died in the delivery room since many of these children were a priori treated in a palliative way. We used random-effects multi-level regression models with centre-specific intercepts to allow for clustering on the study centre level (9 centres) and for year of birth (2000 – 2002; 2003 – 2005; 2006–2008). We then performed multivariate regression analysis for each of the outcome parameters. For multivariate analyses, all explanatory variables that showed an association (p-value <0.10) in the univariate analysis with consistent findings in sensitivity analyses or that are known to act as a confounder were included. The following covariates are included in analyses assessing outcome prediction at birth: gestational age (weeks plus days), birth weight z-score (since birth weight was highly collinear with gestational age), sex, antenatal corticosteroids and multiple birth. The following covariates are included in analyses assessing outcome prediction at 36 weeks PMA: gestational age (weeks plus days), birth weight z-score, sex, antenatal corticosteroids, multiple birth, PDA, NEC, sepsis, ROP stage 3 or higher, BPD, major brain injury and socioeconomic class.

A logistic regression model was calculated to predict adverse and unfavourable outcome after 36^0/7^ weeks PMA. BPD, major brain injury, ROP stage 3 or higher, and NEC or proven sepsis were selected for the model given their strong association with outcomes. The risk for adverse and for favourable outcome was estimated according to whether one, two, three or four of the risk factors were diagnosed in an infant. Associations are given as Odd’s ratios (OR) with 95%-confidence intervals (95% CI) and the two-sided p-values. All analyses were performed using STATA 12.2 (STATA Corporation, College Station, TX, USA) and R for the models with splines.

## Results

### Study population

During the study period, 1326 infants were born alive with a gestational age of 24^0/7^ and 27^6/7^ weeks. Of those, 60 were excluded due to major congenital malformations. Of the included 1266 extremely preterm infants, 422 (33%) infants died: 130 died in delivery room, 280 died after delivery room but before 36^0/7^ weeks PMA, and 12 died between 36^0/7^ weeks PMA and two years of corrected age. Of the 844 surviving infants, 160 (19%) did not receive BSID-II testing and were excluded from the analysis: 109 (13%) infants were lost to follow-up (40 refused follow-up, 53 could not be reached/moved away, 16 unknown loss to follow-up), and 51 (6%) were evaluated using other tests. Children not tested with the BSID-II had a significantly higher gestational age and birth weight, and were more likely to be outborn, delivered by vaginal delivery, and less likely to have BPD than those tested with the BSID-II (Table 1). BSID-II evaluation was thus performed in 684/844 (81%) surviving infants at a median corrected age of 23 months (interquartile range 21 to 25 months). Of the children tested with BSID-II, 440/684 (64%) showed favourable outcome, 166 (24%) moderate ND and 78 (11%) severe ND.

**Table 1:** **Comparison of baseline and neonatal characteristics of infants who had received a BSID**-**II testing versus infants who survived to follow**-**up but who did not receive a BSID**-**II testing**

	**BSID-II(n = 684)**	**No BSID-II(n = 160)**	
	**N (%)**	**N (%)**	**p**-**value**^**§**^
	**Median(IQR)**	**Median(IQR)**	
Inborn	654 (96%)	144 (90%)	0.005
Male gender	362 (53%)	89 (56%)	0.54
Gestational age [weeks]	26.7 (25.9-27.3)	27 (26.1-27.6)	0.007
Birth weight [gram]	855 (730–990)	940 (800–1100)	<0.001
SGA	52 (8%)	7 (5%)	0.15
Singleton	487 (71%)	121 (76%)	0.26
Antenatal corticosteroids (completed)	441 (69%)	99 (65%)	0.29
Caesarean delivery	552 (81%)	117 (73%)	0.028
SES score	6 (4–8)	6 (4–8)	0.72
Umbilical artery pH	7.31 (7.25-7.35)	7.30 (7.23-7.34)	0.17
PDA	311 (46%)	62 (39%)	0.12
PDA ligation	55 (8%)	15 (9%)	0.58
Mechanical ventilation	505 (79%)	109 (73%)	0.14
Mechanical ventilation [days]	4 (1–10)	4 (0–8)	0.20
Bronchopulmonary dysplasia	139 (21%)	18 (11%)	0.008
IVH > 2	46 (7%)	8 (5%)	0.42
Major brain injury	61 (9%)	10 (6%)	0.27
ROP stage 3 or higher	35 (5%)	8 (6%)	0.77
Proven sepsis	171 (25%)	32 (20%)	0.18
Necrotizing enterocolitis	18 (3%)	2 (1%)	0.30

^§^ p-value of Chi-square test (for ratios) and p-value of Mann–Whitney U test (for linear variables); IQR, interquartile range; IVH, intraventricular haemorrhage; NEC, necrotizing enterocolitis; PDA, persistent ductus arteriosus; ROP, retinopathy of prematurity; SES, socioeconomic score; SGA, small for gestational age (<10^th^ percentile).

**Figure 1:**
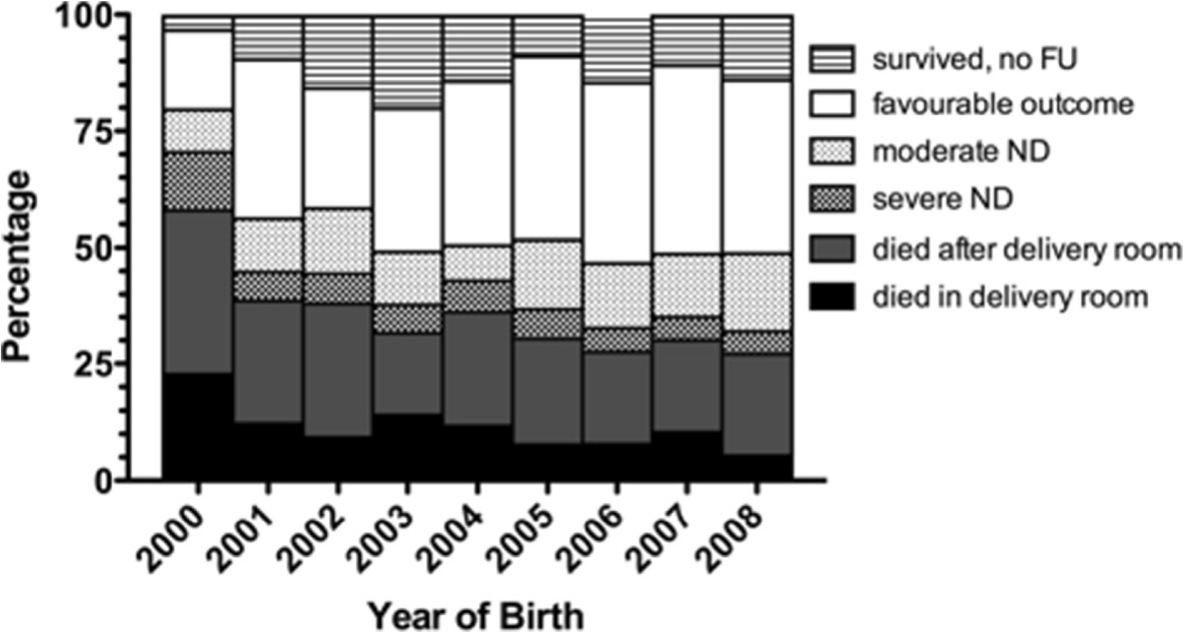
**Changes in mortality and in frequency of severe and moderate neurodevelopmental disability during the study period.** FU, follow-up; ND, neurodevelopmental disability.

Mortality and adverse outcome decreased significantly over the three 3-year observation periods (for mortality: 44%, 32%, 28%; p = 0.01; for adverse outcome 52%, 39%, 33%; p = 0.001; Figure 1). Survival without moderate or severe neurodevelopmental disability increased significantly over the observation periods (27%, 36%, 39%; p = 0.016).

### Outcome prediction at time of birth

**Figure 2:**
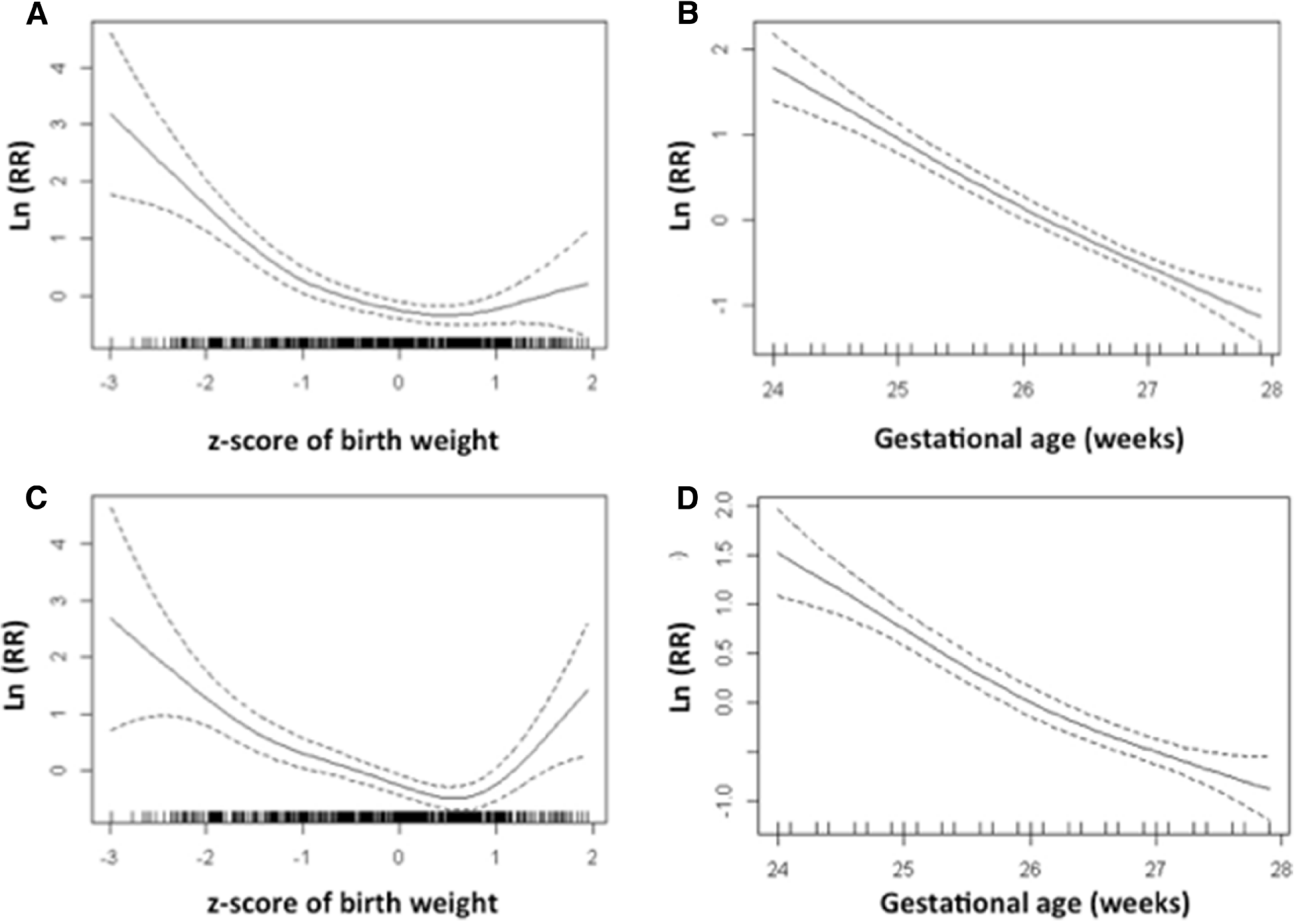
**Spline figures showing non**-**linear associations of gestational age and birth weight with mortality** (**A**, **B**) **and with adverse outcome** (**C**, **D**)**.** The birth weight z-score (**A**, **C**) and the gestational age in weeks (**B**, **D**) are given on the X-axis. Each mark on the X-axis represents a patient. In panel B and D the duration of gestational age was rounded to one decimal. The natural logarithm of the multivariate Odd’s ratio for the respective outcome is shown (y-axis and solid line) with the 95%-confidence intervals (dashed lines).

An overview of the outcomes in relation to the main risk factors is shown in Table 2. Univariate analyses showed that lower gestational age and lower birth weight z-score were significantly associated with mortality, with adverse outcome, and with unfavourable outcome (Table 3). The relationship between gestational age and outcome was linear, whereas the relationship between birth weight and outcome was non-linear with the highest OR in children with a z-score of < −2 (Figure 2). Completion of antenatal corticosteroids for fetal lung maturation induction showed a protective effect on all three outcomes. Multivariate analyses confirmed the strong association of lower gestational age, lower birth weight z-score, and absence of antenatal corticosteroids with all outcomes (Table 3). Male sex was significantly associated with adverse and unfavourable outcome, while in- versus outborn delivery and singleton versus multiple pregnancy were not associated with outcome. In sensitivity analyses excluding infants that died in the delivery room (Table 4), caesarean delivery was not associated with a reduced risk for any of the outcomes.

**Table 2:** **Overview of outcomes in the entire cohort in relation to gestational age**, **birth weight**, **gender**, **and antenatal corticosteroids**

	**Gestational age**									
	**24**^**0/7**^** to 24**^**6/7**^		**25**^**0/7**^** to 25**^**6/7**^		**26**^**0/7**^** to 26**^**6/7**^		**27**^**0/7**^** to 27**^**6/7**^									***Total***										
	**N**	**%**	**N**	**%**	**N**	**%**	**N**	**%**	***N***	**%**																
**Favourable outcome**	28	13	76	28	140	40	196	45	*440*	*35*																
**Moderate ND**	18	9	36	13	51	15	61	14	*166*	*13*																
**Severe ND**	11	5	18	7	21	6	28	6	*78*	*6*																
**Died after delivery room**	61	29	90	33	76	22	65	15	*292*	*23*																
**Died in delivery room**	87	41	28	10	8	2	7	2	*130*	*10*																
**Survived**, **no FU**	5	2	23	8	50	14	82	19	160	13																
Total	210	100	271	100	346	100	439	100	1266	100																
	**Birth weight**																									
	**< 500g**		**500-749g**		**750-999g**		**> 999g**									**Total**										
	**N**	**%**	**N**	**%**	**N**	**%**	**N**	**%**	**N**	**%**																
**Favourable outcome**	4	9	110	27	211	40	115	42	440	35																
**Moderate ND**	5	11	44	11	82	15	35	13	166	13																
**Severe ND**	1	2	25	6	36	7	16	6	78	6																
**Died after delivery room**	22	49	119	29	112	21	39	14	292	23																
**Died in delivery room**	12	27	83	20	25	5	3	1	123	10																
**Survived**, **no FU**	1	2	29	7	65	12	65	24	160	13																
*Total*	*45*	*100*	*410*	*100*	*531*	*100*	*273*	*100*	*1259*	*100*																
	**Sex**																									
	**female**		**male**		***Total***																					
	**N**	**%**	**N**	**%**	***N***	**%**																				
**Favourable outcome**	218	37	222	33	*440*	*35*																				
**Moderate ND**	75	13	91	13	*166*	*13*																				
**Severe ND**	29	5	49	7	*78*	*6*																				
**Died after delivery room**	128	22	164	24	*292*	*23*																				
**Died in delivery room**	64	11	65	10	*129*	*10*																				
**Survived**, **no FU**	71	12	89	13	160	13																				
Total	585	100	680	100	1265	100																				
	**Antenatal corticosteroids**																									
	**incomplete/none**		**completed**		**Total**																					
	**N**	**%**	**N**	**%**	**N**	**%**																				
**Favourable outcome**	140	28	296	41	436	35																				
**Moderate ND**	53	10	113	16	166	13																				
**Severe ND**	24	5	53	7	77	6																				
**Died after delivery room**	139	27	150	21	289	23																				
**Died in delivery room**	95	19	16	2	111	9																				
**Survived**, **no FU**	58	11	99	14	157	13																				
*Total*	*509*	*100*	*727*	*100*	*1236*	*10*																				

Died, patient died in NICU or after discharge from the NICU; died in delivery room, patient died in the delivery room; FU, follow-up; ND, neurodevelopmental disability.

**Table 3:** **Outcome prediction at birth**: **uni**- **and multivariate association of clinical and demographic parameters known at birth with mortality**, **adverse outcome and unfavourable outcome**

**MORTALITY**	**Univariate model†**		**Multivariate model‡**	
	**OR(95%-CI)**	**p**-**value**	**OR(95%-CI)**	**p-value**
**Gestational age** (**weeks**)	0.39 (0.34 - 0.44)	<0.001	0.40 (0.35 - 0.47)	<0.001
**BW z**-**score**	0.64 (0.56 - 0.74)	<0.001	NA	
**BW z**-**score** < −**2**	6.99 (3.09 - 15.85)	<0.001	11.68 (4.73 - 28.86)	<0.001
**BW z**-**score** −**2 to** −**1**	2.10 (1.46 - 3.03)	<0.001	2.71 (11.78 - 4.12)	<0.001
**Male sex**	1.06 (0.82 - 1.35)	0.67	1.15 (0.86 - 1.54)	0.33
**Outborn**	0.80 (0.45 - 1.45)	0.47	NA	
**Antenatal corticosteroids**	0.34 (0.26 - 0.44)	<0.001	0.46 (0.34 - 0.62)	<0.001
**Caesarean delivery**	0.49 (0.37 - 0.65)	<0.001	NA	
**Multiple pregnancy**	1.02 (0.77 - 1.34)	0.90	1.27 (0.91 - 1.78)	0.16
**ADVERSE OUTCOME**	**Univariate model†**		**Multivariate model‡**	
	**OR(95%-CI)**	**p-value**	**OR(95%-CI)**	**p-value**
**Gestational age** (**weeks**)	0.47 (0.41 - 0.53)	<0.001	0.49 (0.42 - 0.56)	<0.001
**BW z**-**score**	0.71 (0.62 - 0.88)	<0.001	NA	
**BW z**-**score** < −**2**	5.36 (2.22 - 12.90)	<0.001	7.90 (3.08 - 20.25)	<0.001
**BW z**-**score** −**2 to** −**1**	1.91 (1.32 - 2.78)	<0.001	2.35 (1.59 - 3.55)	<0.001
**Male sex**	1.20 (0.94 - 1.54)	0.15	1.34 (1.01 - 1.77)	0.042
**Outborn**	0.98 (0.53 - 1.81)	0.95	NA	
**Antenatal corticosteroids**	0.40 (0.31 - 0.53)	<0.001	0.51 (0.38 - 0.69)	<0.001
**Caesarean delivery**	0.53 (0.39 - 0.70)	<0.001	NA	
**Multiple pregnancy**	0.96 (0.73 - 1.27)	0.80	1.19 (0.87 - 1.64)	0.28
**UNFAVOURABLE OUTCOME**	**Univariate model†**		**Multivariate model‡**	
	**OR(95%-CI)**	**p-value**	**OR(95%-CI)**	**p-value**
**Gestational age** (**weeks**)	0.52 (0.45 - 0.59)	<0.001	0.54 (0.47 - 0.61)	<0.001
**BW z**-**score**	0.73 (0.63 - 0.85)	<0.001	NA	
**BW z**-**score** < −**2**	12.05 (2.82 - 51.48)	<0.001	16.91 (3.84 - 74.47)	<0.001
**BW z**-**score** −**2 to** −**1**	1.59 (1.08 - 2.33)	0.019	1.82 (1.20 - 2.74)	0.004
**Male sex**	1.24 (0.96 - 1.59)	0.09	1.35 (1.02 - 1.77)	0.033
**Outborn**	1.30 (0.68 - 2.49)	0.42	NA	
**Antenatal corticosteroids**	0.48 (0.36 - 0.63)	<0.001	0.57 (0.43 - 0.77)	<0.001
**Caesarean delivery**	0.69 (0.51 - 0.93)	<0.001	NA	
**Multiple pregnancy**	1.05 (0.79 - 1.38)	0.76	1.31 (0.96 - 1.78)	0.087

The table gives all parameters that showed an unequal distribution among groups as shown in Table I or that are known from previous publications to act as confounders. † The univariate model is adjusted for study centre and year group. ‡ The multivariate model contains all parameters that were associated with the outcome in the univariate model.

CI, confidence interval; OR, Odd’s ratio; BW, birth weight.

**Table 4:** **Outcome prediction at birth**: **sensitivity analyses excluding infants that died in delivery room** (**n** = **130**): **Uni**- **and multivariate association of clinical and demographic parameters known at birth with mortality**, **adverse outcome and unfavourable outcome**

**MORTALITY**	**Univariate model†**		**Multivariate model‡**	
	**OR(95%-CI)**	**p-value**	**OR(95%-CI)**	**p-value**
**Gestational age** (**weeks**)	0.47 (0.41 - 0.55)	<0.001	0.46 (0.39 - 0.54)	<0.001
**BW z**-**score**	0.66 (0.56 - 0.77)	<0.001	NA	
**BW z**-**score** < −**2**	6.92 (2.89 - 16.59)	<0.001	10.09 (4.01 - 25.40)	<0.001
**BW z**-**score** −**2 to** −**1**	2.02 (1.35 - 3.02)	<0.001	2.52 (1.63 - 3.90)	<0.001
**Male sex**	1.13 (0.86 - 1.49)	0.38	1.16 (0.86 - 1.57)	0.33
**Outborn**	1.15 (0.64 - 2.06)	0.65	NA	
**Antenatal corticosteroids**	0.52 (0.39 - 0.70)	<0.001	0.55 (0.41 - 0.76)	<0.001
**Caesarean delivery**	0.77 (0.55 - 1.06)	0.1	NA	
**Multiple pregnancy**	0.94 (0.69 - 1.29)	0.72	1.28 (0.90 - 1.81)	1.17
**ADVERSE OUTCOME**	**Univariate model†**		**Multivariate model‡**	
	**OR(95%-CI)**	**p-value**	**OR(95%-CI)**	**p-value**
**Gestational age** (**weeks**)	0.56 (0.49 - 0.65)	<0.001	0.55 (0.47 - 0.64)	<0.001
**BW z**-**score**	0.73 (0.62 - 0.85)	<0.001	NA	
**BW z**-**score** < −**2**	4.99 (1.98 - 12.56)	<0.001	6.74 (2.58 - 17.60)	<0.001
**BW z**-**score** −**2 to** −**1**	1.82 (1.22 - 2.72)	0.003	2.20 (1.44 - 3.36)	<0.001
**Male sex**	1.29 (0.98 - 1.69)	0.066	1.35 (1.01 - 1.80)	0.041
**Outborn**	1.34 (0.73 - 2.47)	0.34	NA	
**Antenatal corticosteroids**	0.58 (0.44 - 0.78)	<0.001	0.61 (0.45 - 0.83)	0.002
**Caesarean delivery**	0.79 (0.57 - 1.09)	0.15	NA	
**Multiple pregnancy**	0.91 (0.67 - 1.23)	0.55	1.17 (0.85 - 1.63)	0.34
**UNFAVOURABLE OUTCOME**	**Univariate model†**		**Multivariate model‡**	
	**OR (95%-CI**)	**p-value**	**OR(95%-CI)**	**p-value**
**Gestational age** (**weeks**)	0.60 (0.52 - 0.68)	<0.001	0.59 (0.51 - 0.68)	<0.001
**BW z**-**score**	0.75 (0.64 - 0.87)	<0.001	NA	
**BW z**-**score** < −**2**	11.32 (2.60 - 49.16)	0.001	15.24 (3.43 - 67.71)	<0.001
**BW z**-**score** −**2 to** −**1**	1.47 (0.96 - 2.19)	0.059	1.69 (1.12 - 2.57)	0.013
**Male sex**	1.30 (1.00 - 1.67)	0.049	1.35 (1.02 - 1.78)	0.033
**Outborn**	1.65 (0.87 - 3.15)	0.13	NA	
**Antenatal corticosteroids**	0.64 (0.48 - 0.86)	0.002	0.66 (0.49 - 0.89)	0.006
**Caesarean delivery**	0.96 (0.70 - 1.33)	0.82	NA	
**Multiple pregnancy**	1.01 (0.76 - 1.35)	0.94	1.29 (0.94 - 1.76)	0.11

The table gives all parameters that showed an unequal distribution among groups as shown in Table I or that are known from previous publications to act as confounders. † The univariate model is adjusted for study centre and year group. ‡ The multivariate model contains all parameters that were associated with the outcome in the univariate model.

OR, Odd’s ratio; CI, confidence interval; BW, birth weight.

### Outcome prediction at 36^0/7^ weeks PMA

When restricting the dataset to infants surviving to 36^0/7^ weeks PMA, univariate analyses showed that adverse outcome was associated with male sex, NEC, sepsis, duration of mechanical ventilation, BPD, major brain injury, cystic PVL, and ROP. Unfavourable outcome was associated with lower gestational age, severe growth restriction, sepsis, duration of mechanical ventilation, BPD, cystic PVL, ROP and lower SES class (Table 5).

**Table 5:** **Outcome prediction at 36 weeks gestational age**: **uni**- **and multivariate association of clinical and demographic parameters present by 36 weeks gestational age with adverse outcome**, **and with unfavourable outcome**

**ADVERSE OUTCOME (severe ND or death after 36**^**0**/**7**^**weeks PMA versus favourable outcome or moderate ND)**				
	**Univariate model†**		**Multivariate model‡**	
	**OR(95%-CI)**	**p**-**value**	**OR(95%-CI)**	**p**-**value**
**Gestational age** (**weeks**)	0.81 (0.65 - 1.02)	0.077	1.00 (0.76 - 1.32)	0.98
**BW z**-**score**	0.85 (0.64 - 1.13)	0.271	NA	
**BW z**-**score** < −**2**	1.19 (0.14 - 10.2)	0.88	0.70 (0.08 - 6.33)	0.75
**BW z**-**score** −**2 to** −**1**	1.33 (0.68 - 2.62)	0.41	1.22 (0.56 - 2.67)	0.62
**Male sex**	1.60 (1.00 - 2.54)	0.049	1.35 (0.79 - 2.24)	0.27
**Antenatal corticosteroids**	1.17 (0.70 - 1.96)	0.55	0.86 (0.49 - 1.51)	0.61
**Multiple pregnancy**	0.96 (0.58 - 1.60)	0.88	1.34 (0.77 - 2.36)	0.3
**PDA**	1.52 (0.95 - 2.43)	0.083	1.29 (0.77 - 2.17)	0.33
**NEC**	5.45 (2.25 - 13.22)	<0.001	1.94 (0.58 - 6.50)	0.28
**Sepsis**	1.47 (1.10 - 1.95)	0.008	1.20 (0.87 - 1.65)	0.27
**Duration of ventilation** (**days**)	1.04 (1.02 - 1.06)	<0.001	NA	
**Mechanical ventilation**	3.50 (1.55 - 7.93)	0.003	NA	
**BPD**	3.09 (1.89 - 5.03)	<0.001	2.81 (1.59 - 4.96)	<0.001
**IVH** ≥**3**	1.84 (0.86 - 3.93)	0.11	NA	
**Cystic PVL**	2.07 (1.40 - 3.05)	<0.001	NA	
**Major brain injury**	2.72 (1.45 - 5.10)	0.002	2.64 (1.28 - 5.43)	0.008
**ROP** ≥**3**	2.73 (1.21 - 6.14)	0.015	2.56 (1.05 - 6.26)	0.039
**SES class**	0.74 (0.53 - 1.03)	0.078	0.74 (0.51 - 1.06)	0.096
**UNFAVOURABLE OUTCOME** (**moderate or severe ND or death after 36**^**0**/**7**^**weeks PMA versus favourable outcome**)				
	**Univariate model†**		**Multivariate model‡**	
	**OR** (**95**%-**CI**)	**p**-**value**	**OR** (**95**%-**CI**)	**p**-**value**
**Gestational age** (**weeks**)	0.76 (0.65 - 0.90)	0.001	0.90 (0.74 - 1.10)	0.31
**BW z**-**score**	0.85 (0.70 - 1.04)	0.11	NA	
**BW z**-**score** < −**2**	6.52 (1.26 - 33.79)	0.025	5.29 (0.91 - 30.84)	0.064
**BW z**-**score** −**2 to** −**1**	1.04 (0.63 - 1.73)	0.88	0.95 (0.54 - 1.68)	0.86
**Male sex**	1.35 (0.98 - 1.86)	0.063	1.16 (0.81 - 1.65)	0.42
**Antenatal corticosteroids**	0.97 (0.68 - 1.38)	0.85	0.73 (0.49 - 1.09)	0.12
**Multiple pregnancy**	1.11 (0.78 - 1.58)	0.58	1.31 (0.88 - 1.96)	0.18
**PDA**	1.17 (0.84 - 1.63)	0.35	1.05 (0.73 - 1.53)	0.78
**NEC**	2.34 (0.99 - 5.52)	0.052	1.22 (0.43 - 3.49)	0.71
**Sepsis**	1.26 (1.03 - 1.54)	0.026	1.10 (0.87 - 1.38)	0.42
**Duration of ventilation** (**days**)	1.03 (1.02 - 1.05)	<0.001	NA	
**Mechanical ventilation**	1.23 (0.81 - 1.87)	0.32	NA	
**BPD**	2.26 (1.53 - 3.33)	<0.001	1.92 (1.24 - 2.99)	0.004
**IVH** ≥**3**	1.35 (0.74 - 2.48)	0.33	NA	
**Cystic PVL**	1.43 (1.04 - 1.98)	0.03	NA	
**Major brain injury**	1.68 (0.99 - 2.86)	0.054	1.74 (0.95 - 3.18)	0.07
**ROP** ≥**3**	5.03 (2.33 - 10.86)	<0.001	4.88 (2.07 - 11.52)	<0.001
**SES class**	0.76 (0.61 - 0.96)	0.022	0.74 (0.58 - 0.95)	0.018

The table gives all parameters that showed an unequal distribution among groups as shown in Table I or that are known from previous publications to act as confounders. † The univariate model is adjusted for study centre. ‡ The multivariate model contains all parameters that were associated with the outcome in the univariate model plus demographic factors such as gender and birth weight.

ND, neurodevelopmental disability; OR, Odd’s ratio; CI, confidence interval; PMA, postmenstrual age; BW, birth weight; PDA, persistent ductus arteriosus; NEC, necrotizing enterocolitis ≥ stage 2; BPD, bronchopulmonary dysplasia; major brain injury, intraventricular haemorrhage ≥ grade 3 or cystic periventricular leukomalacia; PVL, periventricular leukomalacia; ROP, retinopathy of prematurity ≥ stage 3; SES, socioeconomic status.

In multivariate models, adverse outcome was significantly associated with BPD, major brain injury and ROP (Table 5). The presence of BPD was the single strongest predictor for adverse outcome (OR 2.81; 95%-CI 1.59 – 4.96; p < 0.001). Unfavourable outcome was significantly associated with BPD, ROP and lower socioeconomic class, whereas major brain injury and severe growth restriction were trend wise associated (Table 5). The presence of ROP stage 3 or higher was the single strongest predictor for unfavourable outcome (OR 4.88; 95%-CI 2.07 – 11.52; p < 0.001).

### Prediction model of outcome using count of major neonatal morbidities

**Figure 3:**
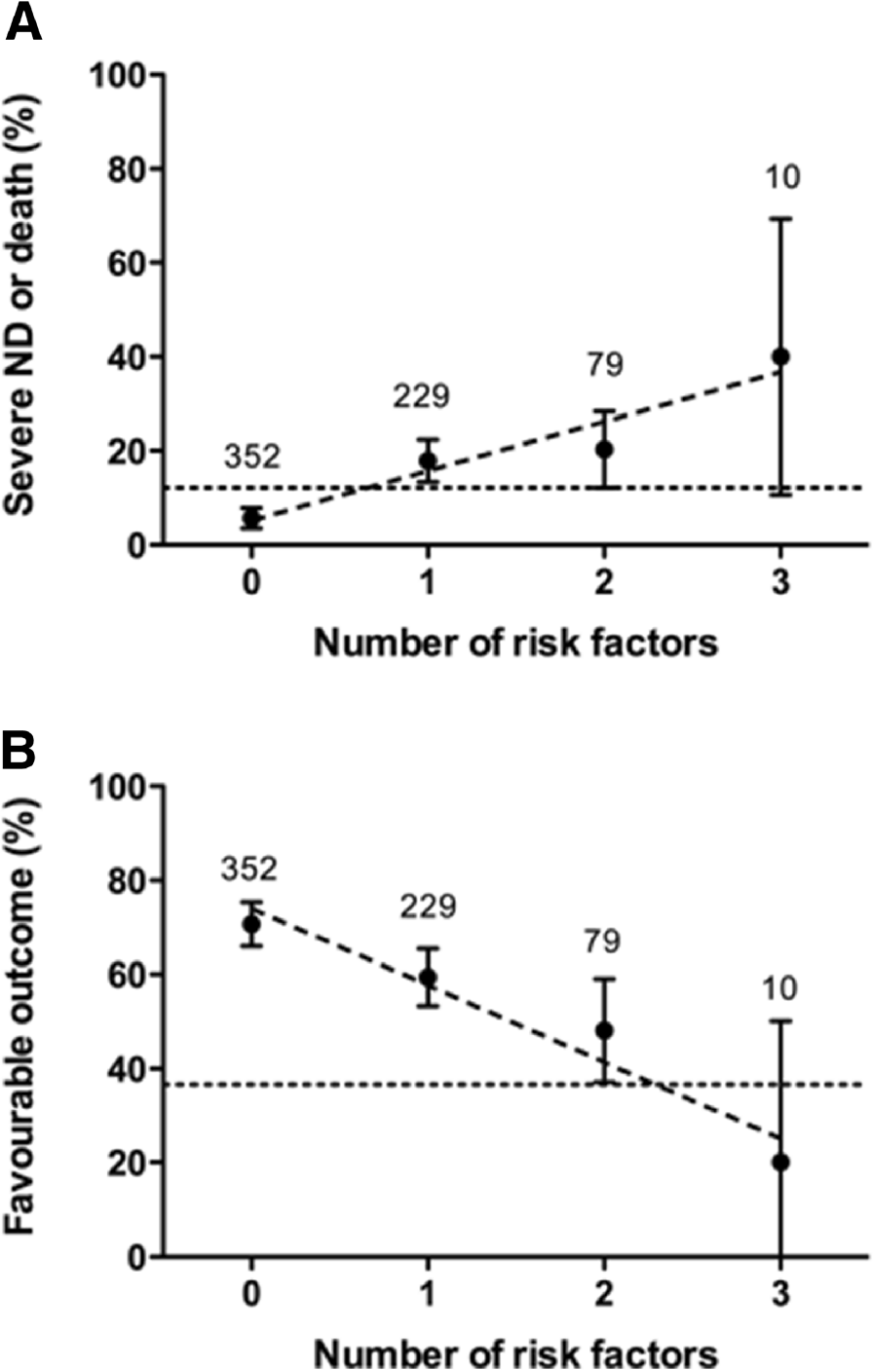
**Model of prediction of outcome according to count of four risk factors** (**bronchopulmonary dysplasia**, **major brain injury**, **retinopathy of prematurity stage 3 or higher**, **and necrotizing enterocolitis and**/**or proven sepsis**). The proportion of adverse outcome (**A**) and of favourable outcome (**B**) with 95%-confidence intervals and the number of infants per group are shown. The dotted horizontal line indicates the average proportion of the respective outcome. The dashed diagonal line represents the regression line. ND, neurodevelopmental disability. The figure shows data from 670 children because information on risk factor ROP (stage 3 or higher) was not available in 14 of 684 patients.

The risk for adverse and for unfavourable outcome increased strongly with each addition of a major neonatal risk factor (BPD, major brain injury, ROP stage 3 or higher, NEC or proven sepsis), with a p-value of <0.001). Infants with none of the risk factors had an adverse outcome in 6% and had a favourable outcome in 71% (Figure 3). Infants with three risk factors had an adverse outcome in 40% and a favourable outcome in 20%.

## Discussion

This study reports on mortality and outcome at two years of age in extremely preterm infants in a Swiss cohort born after 2000, based on a nationwide prospective registry and follow-up program. Thereby it provides representative outcome information on a national level over a nine-year period. To the best of our knowledge, these data represent one of the largest datasets on a recent birth cohort.

The incidence of severe ND was 11%, while 24% of infants survived with moderate ND. The overall rate of any ND was moderately higher than reported in a similar cohort born in 2005 in Victoria, Australia [28]. Mortality depended strongly on gestational age and decreased from 70% at 24 weeks to 17% at 27 weeks. The observed mortality rates were slightly higher compared to a U.S. cohort [29] and a recent Australian cohort [28], but similar to European data [30]. We observed a significant decrease in mortality and severe ND over the study period with a parallel increase in survival without moderate or severe neurodevelopmental disability. This trend is supported by the observed overall decrease in major neonatal morbidities in preterm infants during the last decade in Switzerland [31]. However, some caution is required when interpreting the observed trend since implementation of BSID-II increased between 2000 and 2005 in the participating centres. We therefore adjusted analyses using random multilevel clustering for each centre in order to account for the effect of centre-to-centre differences. Since this is a purely observational cohort on neonatal morbidities and outcomes, it is not possible at this stage to identify specific changes in management that potentially could have led to improved outcomes over time. Theoretically, a variety of factors such as improved prenatal care, early start of CPAP, optimal feeding and strategies to reduce nosocomial infection may contribute to improved outcomes. The publication of the Swiss guidelines [32] for the care of infants between 22 and 26 gestational weeks in 2002 may have affected the decision to provide palliative versus intensive care in infants below 26 weeks GA. Therefore, this study was based only on infants born after at least 24 0/7 weeks PMA and we performed sensitivity analyses excluding infants that died in delivery room. The sensitivity analyses confirmed the main results.

At birth, death and severe ND were mainly predicted by low gestational age and low birth weight, while antenatal corticosteroids to induce fetal lung maturation had a strong protective effect. Male sex increased the risk of adverse outcome, but it played a minor role compared to previous reports [12,13]. Surprisingly, outborn and multiple birth status did not affect outcome prediction in our cohort, but the rate of outborns was small. In the past, the majority of publications reporting on neonatal outcomes used both gestational age and birth weight as covariates, despite the strong collinearity between these. We think our model is more accurate, since birth weight is not an independent variable, but a result of gestational age and intrauterine growth (z-score of birth weight). Multivariate analyses confirmed that the z-score of birth weight was strongly associated with outcomes. Whether caesarean delivery has a protective effect is debated in the literature [33,34]. In our study, delivery by caesarean delivery showed a protective benefit in the univariate risk analysis, but this effect disappeared in the sensitivity analyses, suggesting the association was strongly confounded by the antenatal decision to treat a child in a palliative way and thus avoiding caesarean delivery. Additional multivariate analyses including delivery mode as a covariate resulted highly similar to the main models and caesarean delivery was not significantly associated with any of the outcomes (data not shown).

Long-term outcome in children surviving to 36^0/7^ weeks PMA was only weakly influenced by the main perinatal factors gestational age, intrauterine growth, sex and lung maturation. Instead, the major neonatal morbidities such as BPD, ROP, major brain injury and NEC or sepsis played a major role. Our results are in agreement with a previous study reporting that the prognosis for very preterm infants changes during the course of early postnatal life depending on the incidence of neonatal morbidities [35]. Our findings confirm that antenatal corticosteroids improve survival without increasing morbidity in the survivors [36]. NEC and sepsis were strongly associated with outcomes in univariate analyses, which is in agreement with previous studies [10,22]. However, in multivariate models, BPD was the single strongest predictor of adverse outcome, while ROP, followed by BPD, was the strongest predictor of unfavourable outcome. Interestingly, further analyses revealed that sepsis was one of the strongest risk factors for BPD (multivariate p = 0.006), together with low gestational age, intrauterine growth restriction and absence of antenatal corticosteroids. This indicates that while BPD in our model is the better predictor of adverse outcome, sepsis represents one of the main causative factors leading to BPD. It is important but often difficult to distinguish whether a covariate is a marker of disease severity or actually a factor causing poor outcome. Mechanical ventilation and sepsis are known to cause direct damage to the lungs and, indirectly, to the brain [37].

Several limitations of this study need to be mentioned. Nineteen percent of surviving infants did not receive BSID-II testing and were excluded from the logistic regression analyses. Even if our follow-up rate of 81% is comparable to published studies [38], the infants lost from follow-up displayed a higher GA and a lower BPD rate than the study group. We cannot exclude that the loss from follow-up of these infants may have led to a slight underestimation of the overall outcome. We decided a priori to predict outcome at two time points, once at time of birth and once at 36 weeks gestational age. The selection of these two time points makes sense from a clinical point of view, since outcome prediction is most important pre-delivery and when approaching discharge. However, this model does not allow assessing the impact of the main postnatal morbidities on early neonatal mortality. Since data on chorioamnionitis and postnatal corticosteroids therapy were not prospectively recorded, we cannot comment on the impact of these two factors [39,40] on outcome. Finally, outcome assessment at two years of age may both over - and underestimate long-term cognitive and motor neurodevelopment [41].

The strengths of this study include the fact that it is based on a large national prospective database. The definition of neurodevelopmental outcomes was based on a recent consensus definition. The definition of severe ND required extremely low cognitive or motor performances, i.e. <−3SD, and/or severe neurosensory disabilities, thus identifying severely disabled children. In comparison, the definition of neurodevelopmental disability used in several previous studies overlaps with our definition of severe and moderate ND [11,22,42]. The uni- and multivariate statistical models were highly similar, confirming the independent contribution of the major risk factors on outcome.

## Conclusions

This population-based study of extremely preterm infants shows that, although more than a third of infants still suffer from moderate or severe ND, the rate of survival without major neurologic sequelae has increased significantly over the last decade, while the mortality rate decreased. Neonatal mortality was predominantly dependant on gestational age, birth weight z-score and antenatal corticosteroids, while outcome at two years of age in children surviving the neonatal period was best predicted by BPD, ROP, and major brain lesions as diagnosed using head ultrasound examination. This study provides representative national data, which may assist in decisions on care of extremely preterm infants, and which can contribute to improve both the quality of care as well as the counselling of parents.

## Competing interests

Giancarlo Natalucci received financial support by the Swiss National Science Foundation; grant 33CM30-124101. The authors declare no actual or potential conflict of interest in relation to this manuscript.

## Authors’ contributions

LS, MA, and GN had primary responsibility for the study design, data acquisition, data analysis and writing the manuscript. EP was involved in data analysis and data interpretation and writing of the manuscript. MA, MAe, BL, HUB, SG, CBT, and MBG were involved in study design, data acquisition and in revising the manuscript. All authors approved the final version of this manuscript.

## Pre-publication history

The pre-publication history for this paper can be accessed here:

http://www.biomedcentral.com/1471-2431/12/198/prepub
